# The effect of systematic exercise training on skeletal muscle strength in a patient with advanced inclusion body myositis: A case study

**DOI:** 10.17159/2078-516X/2022/v34i1a13145

**Published:** 2022-01-01

**Authors:** C D’Alton, R Johnstone, C du Plessis, A Pursad, T A Kohn

**Affiliations:** 1 HPALS, Division of Physiological Sciences, Department of Human Biology, University of Cape Town, Cape Town, South Africa; 2 Sports Science Institute of South Africa, Boundary Road, Newlands, Cape Town, 7725, South Africa; 3 Department of Medical Bioscience, Faculty of Natural Sciences, University of the Western Cape, Bellville, South Africa

**Keywords:** myopathy, autoimmune disease, muscle weakness, resistance training

## Abstract

Inclusion body myositis (IBM) is an inflammatory and degenerative autoimmune disease that targets specific muscle groups, causing severe muscle weakness. Exercise training is often contraindicated in myopathies as it may aggravate muscle damage and inflammation. Although some reported positive outcomes in muscle strength of early diagnosed IBM patients undergoing resistance training, there remains uncertainty as to whether exercise could be beneficial and safe in advanced stage IBM. Thus the aims of this research were to evaluate the safety and response of 16-weeks supervised resistance training on the health and muscle performance of an elderly participant diagnosed with advanced stage IBM. It was shown that the training had no adverse effects on the health of the patient. Muscle strength measured at eight weeks and on completion of the intervention, remained the same as at baseline. In conclusion, the exercise programme was found to be safe and seemed to maintain muscle strength in a patient with advanced stage IBM.

Inclusion body myositis (IBM) is an acquired autoimmune myopathy with no known cause or cure. ^[1]^ Clinically, the disease presents with a slow progressive decline in muscle strength, resulting from muscle fibre atrophy and the destruction of the quadriceps, forearm flexors and ankle dorsiflexion muscles as result of an inflammatory degenerative process within skeletal muscle, although the primary pathological process remains unclear.^[1]^ Histological sections of the muscle show inflammation of non-necrotic muscle fibres, with amyloid deposits (inclusions) evident within the vacuoles. IBM is initially difficult to diagnose as the first diagnosis presents as any inflammatory myopathy, with the inclusions only appearing much later.^[1]^ There is currently no effective treatment for IBM.

Studies using endurance and resistance training have been shown to benefit the physical and psychological wellbeing of patients with IBM.^[2–4]^ Resistance training improves muscle strength through increased muscle fibre recruitment and hypertrophy and is therefore a viable and inexpensive method to aid and maintain muscle strength in IBM patients.^[4]^ South Africa currently has no standard exercise intervention programmes specifically designed towards improving muscle function in patients with myopathies, partly due to the conventional belief that exercise may cause more muscle damage. However, studies on IBM and other myopathies (e.g. McArdle disease) are proving that supervised exercise training is a viable adjunct in maintaining or improving muscle strength.^[4,5]^

The aims of this study were to (i) evaluate the safety and (ii) the response of a 16-week supervised resistance training exercise protocol on the health and physical muscle performance of an elderly patient diagnosed with advanced stage IBM. The outcomes of this study may be applied more broadly to other myopathy cases.

## Case report

### History

The patient was a 71-year-old male in good health, despite having advanced stage IBM, of which clinical symptoms (muscle weakness) were already presented in November 2006. Due to the clinical overlap between polymyositis and the earlier stage of IBM, the diagnosis of IBM was only confirmed on a muscle biopsy in October 2016.

At the time of the exercise intervention (two years later), the patient was able to walk with the aid of a three-wheel walker but was unable to stand up from a chair by himself. During his functional assessment he was also found to have severe hyperextension of his knees.

Despite his physical limitations as a result of the IBM and the use of the following chronic medications, which included 100 mg/day allopurinol, 7.5 mg zopiclone and 25 mg amitriptyline at night, 0.4 mg/day tamsulosin, 5 mg/day folic acid, 300 mg/day irbesartan and 12.5 mg/day hydrochlorothizide, he was classified as healthy and cleared to take part in the exercise training intervention.

### Methods

The Faculty of Health Sciences Research Ethics Committee of the University of Cape Town (HREC 089/2018) approved the study and the participant provided his written informed consent. The protocol consisted of physical, clinical and fitness assessments, including the acquisition of blood samples. On completion of the above tests, the participant underwent eight weeks of exercise training. The assessments were then repeated, followed by a second exercise training period of eight weeks, and a final assessment period on completion of the exercise intervention.

### Clinical assessment

Prior to participation in the exercise sessions, the participant underwent a clinical review to determine his current physiological and psychological health status. This included the completion of the Barthel Index of Activities for Daily Living questionnaire, the Fatigue Severity Scale, as well as the Visual Analogue Fatigue Scale to evaluate fatigue severity, all of which were also repeated at week eight and 16.

The clinical assessment entailed obtaining a complete medical history, including current medications, followed by a thorough routine clinical examination with specific emphasis on the cardiorespiratory, renal and neuromuscular systems. These were repeated midway through the programme. Included in the assessment was a urine sample analysis using urinary dipsticks to exclude any baseline abnormalities, such as glucosuria, evidence of an UTI or underlying renal disease, but most importantly, the presence of myoglobinuria (Combur testing kit, Roche). The rationale for this specific investigation was to assist in the monitoring for the presence of myoglobinuria as an additional method of monitoring for excessive muscle damage (rhabdomyolysis) during the exercise intervention phase if he was to present with Delayed Onset Muscle Soreness (DOMS) or dark-coloured urine which was not found throughout the intervention. The patient was deemed healthy and the exercise was not considered to pose any adverse risks to him.

### Exercise test assessments

Isokinetic strength tests of the knee and elbow joints were conducted using a dynamometer (Biodex Medical Systems, Inc., NY, USA) before, during and after the exercise training programme (presented in Table 1). Handgrip strength was measured bilaterally using a hand-held dynamometer (Camry Electronic Hand Dynamometer EH101, Camry Scales, CA, USA). A blood sample for creatine kinase (CK) level determination was obtained a day after the exercise test to assess muscle damage both before the start of the intervention as well midway, because the exercise tests were considered to be the greatest risk for inducing muscle damage. The tests were not repeated after the final assessment due to the patient’s stable clinical nature.

### Resistance training protocol

Exercise sessions (one hour each) were supervised by the same two qualified biokineticists, and consisted of moderate-intensity resistance exercises (isometric and isotonic) using rubber bands, three times per week performed for 16 weeks. Intensity was monitored using the Rating of Perceived Exertion (RPE) scale (rate of 1 to 10) which was maintained between the scale of 7 and 8. Whenever an exercise was too easy or difficult, adjustments to the programme were made. The patient was also closely monitored for any DOMS.

## Discussion

At baseline, ankle plantar flexion, wrist flexion and wrist extension were all within the normal range for the participant’s age category conforming to the muscles rarely affected by IBM.^[1,6]^ Interestingly, elbow extension appeared in the normal range and its strength was not affected by the disease. Knee flexion and extension, ankle dorsiflexion and elbow flexion were severely compromised due to the IBM and well below the normal range. The muscles of the right- and left- sided limbs were found to be similarly affected by the disease, but grip strength was consistently greater in the left hand. However, this was considered to be weak when compared to the age-related ranges (Table 1).

The participant tolerated and complied well with the maximum exercise tests and training. He presented with no adverse side effects, besides the reporting of some stiff limbs due to the training. Serum CK was measured (Pathcare, Cape Town, South Africa) a day after the initial and submaximum exercise tests (188 and 181 IU/l, respectively), concluding that muscle damage was minimal. Overall, the data showed no significant change (improvement or deterioration) in the isokinetic strength of the participant, but variable responses between different muscle actions were noted for which the exact mechanisms are unclear. What is also unclear is whether the exercise programme may have aided in the maintenance of muscle strength. A comparison of strength changes following a trial period without exercise would have been necessary to make such a conclusion.

However, the participant’s self-reported scores for the Fatigue Severity Scale and Visual Analogue Fatigue Scale tests improved from 31 to 28 and from 5 to 8, respectively, over the course of the exercise intervention, while his Barthel score remained unchanged, suggesting a self-perceived reduction in his susceptibility to fatigue. The participant’s additional perceived benefits included better sleep patterns and mobility in bed, less achy muscle and joints, better distal perfusion, improved balance on standing, improved quadriceps, biceps and triceps strength, less over-extension of his left knee, and the ability to cover longer distances with his 3-wheel walker. He was still unable to rise unaided from a seated position or climb stairs, and he felt that his gluteal and hamstring muscles had showed no increase in strength. A muscle biopsy from the vastus lateralis before and after training did form part of the study’s design to investigate any changes that might have occurred from the training, but was unsuccessful due to the severe muscle atrophy and wasting.

Some previous research on exercise training in IBM patients reported improvements in muscle strength, whereas others showed no change.^[3,4]^ It is important to note that the patient from the present study was severely affected by the disease, whereas most of the other reported studies excluded patients with such disease severity and thus the responses may have been different.

## Conclusion

The data from this case study showed that systematic supervised resistance exercise was safe in an advanced staged IBM patient. The training did not appear to have improved muscle strength, but could have resulted in maintained muscle function.

## Figures and Tables

**Table 1:** Maximum physical performance markers measured during the three assessments

	Baseline assessment			Week 8 assessment			Week 16 assessment			Normal range for 70 – 83 age group

Isometric contractions (N·m)	Left	Right	Mean	Left	Right	Mean	Left	Right	Mean	
Knee flexion	10	11	**11**	12	12	**12**	13	10	**11**	24 – 38
Knee extension	11	10	**11**	9	13	**11**	9	12	**11**	55 – 71
Ankle plantar flexion	41	42	**42**	31	30	**31**	43	22	**33**	25 – 46
Ankle dorsiflexion	Could not initiate exercise			Could not initiate exercise			Could not initiate exercise			12 – 13
Elbow flexion	6	7	**6**	4	7	**6**	1	8	**5**	14 – 19
Elbow extension	15	18	**17**	15	22	**18**	16	25	**21**	14 – 23
Wrist flexion	5	6	**6**	7	7	**7**	9	9	**9**	6 – 9
Wrist extension	9	18	**14**	6	10	**8**	11	8	**10**	4 – 5

**Grip strength (kg)**	27	6	**16**	17	6	**11**	21	8	**15**	28 – 42

Isometric contractions values are presented as torque (Newton·metres), whereas grip strength is reported in kilograms (kg). Left indicates flexion or extension of the left limb; Right indicates flexion or extension of the right limb.
